# Supplementary material for: Incidence and predictors of postpartum depression among postpartum mothers in Kuala Lumpur, Malaysia: A cross-sectional study
Source: PLoS One. 2021 Nov 9;16(11):e0259782. doi: 10.1371/journal.pone.0259782 (PMC8577760; doi:10.1371/journal.pone.0259782)
Supplement: S1 Table — (DOCX) [file pone.0259782.s001.docx]

**S1 Table.** **Socioeconomic characteristics of the participants (N=350) in this study**

| **Socioeconomic characteristics** | **N** | **%** |
| --- | --- | --- |
| Ethnicity   - Malay - Chinese - Indian - Others | 306  23  13  8 | 87.43  6.57  3.71  2.29 |
| Postpartum period (months)   - 1 - 2-3 - 4-6 | 197  110  63 | 56.20  31.43  18.00 |
| Number of children   - 1 - 2 - 3 and above | 125  104  121 | 35.70  29.70  34.60 |
| Monthly household income   - Low (<MYR4360) - Middle (MYR4360 to MYR9619) - High (≥MYR9619) | 168  163  19 | 48.00  46.60  5.40 |
| Maternal education status   - Low (Up to secondary school) - Medium (Up to pre-university/diploma) - High (Completed tertiary education) | 105  130  115 | 30.00  37.10  32.90 |
| Maternal employment status   - Employed - Self-employed - Housewife/homemaker | 229  39  82 | 65.40  11.10  23.40 |
| Living arrangements   - Own house - With participant’s extended family - With spouse’s extended family | 257  38  55 | 73.43  10.86  15.71 |
| Birth location   - Government hospital - Private hospital - Own house | 313  36  1 | 89.40  10.30  0.30 |
