# Supplementary material for: Incidence and predictors of postpartum depression among postpartum mothers in Kuala Lumpur, Malaysia: A cross-sectional study
Source: PLoS One. 2021 Nov 9;16(11):e0259782. doi: 10.1371/journal.pone.0259782 (PMC8577760; doi:10.1371/journal.pone.0259782)
Supplement: S2 Table — (DOCX) [file pone.0259782.s002.docx]

**S2 Table. Postpartum depression (PPD) incidence based on the study participants’ socioeconomic characteristics & PPD literacy**

| **Participants’ characteristics** | **PPD incidence** | | | | ***Χ^2^* or *t*** | ***p*-value** |
| --- | --- | --- | --- | --- | --- | --- |
|  | **Yes (n=50)** | | **No (n=300)** | |  |  |
|  | **n** | **%** | **n** | **%** |  |  |
| Mean age | 29.02±5.44 | | 30.84±4.54 | | 2.55 | 0.03 |
| Mean PoDLiS score | 3.68±0.38 | | 3.78±0.37 | | 1.90 | 0.06 |
| Ethnicity  • Malay  • Chinese  • Indian  • Others | 40  4  3  3 | 15.04  21.05  30.00  60.00 | 266  19  10  5 | 84.96  78.95  70.00  40.00 | 4.89 | 0.18 |
| Postpartum period (months)  • 1  • 2-3  • 4-6 | 33  10  7 | 18.80  11.10  11.10 | 164  80  56 | 83.20  88.90  88.90 | 2.24 | 0.33 |
| Number of children in the family  • 1  • 2  • 3 and above | 25  10  15 | 20.00  9.60  12.40 | 100  94  106 | 80.00  90.40  87.60 | 5.54 | 0.06 |
| Monthly household income  • Low  • Middle  • High | 36  14  0 | 27.27  8.60  0.00 | 132  149  19 | 78.60  91.40  100.00 | 14.49 | 0.01 |
| Maternal education status  • Low  • Medium  • High | 18  17  15 | 17.10  13.10  13.00 | 87  113  100 | 82.90  86.90  87.00 | 0.97 | 0.61 |
| Maternal employment status  • Employed  • Self-employed  • Housewife/homemaker | 27  4  19 | 13.37  11.43  30.16 | 202  35  63 | 86.63  88.57  69.84 | 6.97 | 0.03 |
| Living arrangements  • Own house  • With participant’s extended family  • With husband’s extended family | 35  6  9 | 15.77  18.75  19.57 | 222  32  46 | 84.23  81.25  80.43 | 0.35 | 0.55 |
| Birth location  • Government hospital  • Private hospital  • Own house | 48  2  0 | 15.30  5.60  0.00 | 265  34  1 | 84.70  94.40  100 | 2.69 | 0.26 |
