# Supplementary material for: Incidence and predictors of postpartum depression among postpartum mothers in Kuala Lumpur, Malaysia: A cross-sectional study
Source: PLoS One. 2021 Nov 9;16(11):e0259782. doi: 10.1371/journal.pone.0259782 (PMC8577760; doi:10.1371/journal.pone.0259782)
Supplement: S3 Table — aThe reference category for ethnicity is the Other ethnic groups. bThe reference category for monthly household income is the middle- to high-income group (≥MYR4360). cThe reference category for maternal employment status is unemployed. *significant at p<0.05 **significant at p<0.01. (DOCX) [file pone.0259782.s003.docx]

**S3 Table**. **Binary logistic regression analysis**

| **Predictors** | **Unadjusted model** | | **Adjusted model** | |
| --- | --- | --- | --- | --- |
|  | **Odds ratio [95% CI]** | **P-value** | **Odds ratio**  **[95% CI]** | ***p*-value** |
| Participant’s age | 0.92 [0.86, 0.98] | 0.01* | 0.94 [0.88-1.00] | 0.07 |
| Monthly household income  • <MYR4360  • ≥MYR4360^b^ | 3.27 [1.70, 6.31]  1 | <0.001** | 2.58 [1.23, 5.19]  1 | 0.01* |
| Maternal employment status  • Employed  • Self-employed  • Housewife/homemaker^c^ | 0.44 [0.23, 0.85]  0.38 [0.12, 1.20]  1 | 0.01*  0.10 | 0.64 [0.32, 1.28]  0.51 [0.16, 1.66]  1 | 0.21  0.27 |

^a^The reference category for ethnicity is the Other ethnic groups

^b^The reference category for monthly household income is the middle- to high-income group (≥MYR4360)

^c^The reference category for maternal employment status is unemployed

*significant at p<0.05 **significant at p<0.01
